# Knock-down of BCL6 / STAT6 sensitizes primary B cell lymphoma cells for treatment with current therapeutic agents

**DOI:** 10.18632/oncoscience.35

**Published:** 2014-04-30

**Authors:** Marie-Therese Häberle, Elena Kelsch, Karola Dorsch, Peter Möller, Olga Ritz

**Affiliations:** ^1^ Helmholtz Zentrum, Munich, Germany; ^2^ Institute of Pathology, University of Ulm, Germany

**Keywords:** Doxorubicin, Rituximab, Vincristin, PMBL

## Abstract

Primary mediastinal B cell lymphoma (PMBL) is characterized by specific molecular hallmarks including the expression of B Cell Lymphoma factor 6 (BCL6) and the presence of the activated Signal Transducers and Activators of Transcription factor 6 (STAT6). Recently we have shown that combined targeting of BCL6 and activated STAT6 by specific chemical inhibitors in PMBL resulted in additive efficacy regarding their negative effects on cell viability. Given that despite general efficient immuno-chemotherapy in PMBL the delayed treatment-related sequelae remains still a main challenge, we analyzed the role of BCL6 and activated STAT6 in the sensitivity of PMBL cells to the current treatment components. We found that the knock-down of BCL6 / STAT6 by siRNA sensitized the PMBL cells to the effects of R-CHOP components in two of three PMBL cell lines. In one cell line, MedB-1, which is marked by less expression of BCL6 and mutated STAT6, the knock-down of BCL6 / STAT6 did not enhance the efficiency of Doxorubicin, Rituximab, and Vincristin.

Thus, the targeting of BCL6 and STAT6 in addition or prior to the treatment with components of the current immuno-chemotherapy may sensitize the PMBL tumor cells for drug effects, at least in parts of PMBL cases.

## INTRODUCTION

Primary mediastinal B cell lymphoma (PMBL) is a distinct subtype of aggressive B cell lymphoma and meanwhile belongs to the most curable lymphoma subtypes [1]. Since relapses after first-line therapies usually occur within the first 12 months and salvage therapies have been reported to have high failure rates, the successful primary treatment has been considered decisive. In this context even more dose-intense regimens have been suggested although the main goal still is to minimize treatment-related sequelae [2]. Therefore, further investigations of possible specific molecular targets which may improve standard R-CHOP are warranted.

One of specific molecular characteristics of PMBL is the presence of the anti-apoptotic and pro-proliferative factors BCL6 in about 90% [3-5] and pSTAT6 in about 70% of cases [6], respectively. Recently we have shown that the inhibition of these two factors by using chemical compounds may open a new therapeutic approach in PMBL [7]. The intratumoral distribution of these two factors in PMBL is very heterogeneous. The neoplastic cells express BCL6 in very variable numbers ranging from 10-90%; whereas the percentages of pSTAT6-positive cells vary between 0-14 [7]. Thus, chemical targeting these two factors would only reach subpopulations of tumor cells. Nevertheless, the combination of the standard therapy with the specific targeting of BCL6 or activated STAT6 may be of advantage in PMBL.

Here we used knock-down approach to evaluate the role of BCL6 / STAT6 in the modulation of the PMBL cell sensitivity to the commonly used therapeutic agents, such as Doxorubicin, Rituximab, and Vincristin which represent major components of the current standard therapy for PMBL, R-CHOP (Rituximab, Cyclophosphamide, Doxorubicin, Vincristine, and Prednisone). We found in 2/3 PMBL cell lines that downregulation of BCL6 / STAT6 sensitizes the tumor cells for treatment with immuno-chemotherapeutic agents.

Therefore, our study suggests that targeting BCL6 / STAT6 additionally to standard therapy in PMBL may further improve responsiveness of these tumors.

## RESULTS AND DISCUSSION

### Experimental design

**Figure 1:**
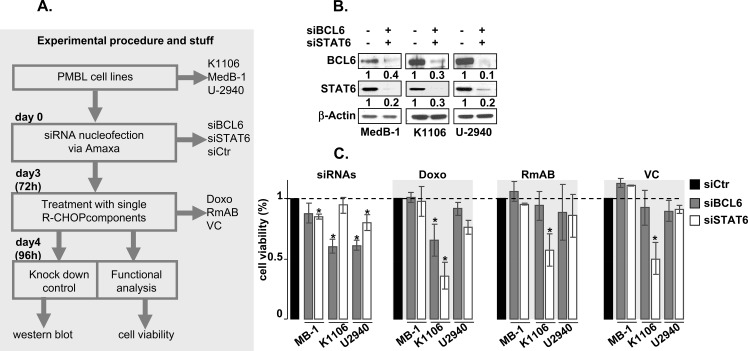
The functional analysis of BCL6 / STAT6 role in the sensitization of PMBL cells to current therapeutic agents **(A)** Experimental design **(B)** western blot showed the knock-down of BCL6 or STAT6 in three PMBL cell lines: MedB-1, K1106, U-2940. Beta-actin was used as a loading control. The ratio of BCL6 or STAT6 protein in samples were treated either with specific siRNA (siBCL6 or siSTAT6) or with control siRNA as indicated. **(C)** Cell viability assay in PMBL cell lines by using luminescence assay. The black bars represent the sample transfected with control siRNA (siCtr) and were used as a reference for the calculation of the cell viability in samples where BCL6 (siBCL6) or STAT6 (siSTAT6) were downregulated. The dotted line represents the amount of viable cells corresponding to that of siCtr samples. All provide as averages ± standard error of the mean (SEM) relative to siCtr sample. The results from three independent experiments are presented and p-value indicators * are provided when *p*<0.05.

In order to investigate the potential role of BCL6 and pSTAT6 in the modulation of PMBL sensitivity to the anti-tumor effects of Doxo, RmAb and VC we followed the experimental scheme shown in Figure 1A. First, we transiently transfected three PMBL cell lines, MedB-1, K1106, and U2940, with either siRNA targeted BCL6 (siBCL6) / STAT6 (siSTAT6), or non-specific control siRNA (siCtr) and used Amaxa nucleofection method.

After 72h of post-transfection the therapeutic agents, Doxorubicine (Doxo), Rituximab (RmAB), and Vincristin (VC) were added and cells were incubated for further 24h. In each cell lines the efficacy of BCL6 or /and STAT6 downregulation mediated by siBCL6 or siSTAT6 relative to control sample (siCtr) was monitored by western blot (Figure 1B).

The cell viability analysis was performed by using a luminescence method based on quantitation of the ATP as the measure for metabolically active cells.

### Control of knock-down of BCL6 / STAT6 by siRNA

As showen in Figure 1B, the knock-down of BCL6 in three PMBL cell via siRNA resulted in a clear reduction of BCL6 protein down to 30% in K1106, 10% in MedB-1, and 40% in U2940. The knock-down of STAT6 was even more efficient and the remaining STAT6 protein was 30% in K1106; 20% in MedB-1; 20% in U2940. The calculation of the remaining BCL6 and / or STAT6 protein was done relative to loading control β-actin.

### Functional analysis

Next, we analysed the cell viability by a luminescence assay, based on the quantitation of the ATP as a measure of metabolically active cells. We observed that downregulation of BCL6 or STAT6 in K1106 and U2940 sensibilized the cells to the anti-tumor effects of Doxo, RmAB, and VC by impairing cell viability, although some differences between cell lines were detected (Figure 1C). Notably, the supportive effects of STAT6 knock-down were mostly superior to that of BCL6 knock-down in K1106 and U2940 (compare the grey and white bars for K1106 and U2940 in Figure 1C).

These results underline our recently published data regarding the new therapeutic approaches using chemical inhibition of BCL6 and /or STAT6 [7]. Thus, the combination of current immuno-chemotherapy with BCL6 / STAT6 downregulation may be an advantageous for PMBL patients.

Unexpectedly, in MedB-1 cells neither the knock-down of BCL6 nor of STAT6 induced any additional anti-tumor effects to the threatment with Doxo, RmAB, and VC (Figure 1C). Moreover, the reduction of cell viability mediated by RmAB and VC was even slightly diminished in MedB-1 samples with downregulated BCL6 / STAT6.

The expression of BCL6 in MedB-1 cells is less frequent then in the other two PMBL cell lines (~ 10% cells in MedB-1, compared to 40% in K1106 and 90% in U2940) [7]. Of note, the knock-down of BCL6 by siRNA did not have significant effects on MedB-1 cell survival (Figure 1C, left panel). Nevertheless, the treatment with specific BCL6 inhibitor 79-6 [9] resulted in a clear decrease of cell viability also in MedB-1, although after removal of this inhibitor MedB-1 cells showed the highest recover rate [7]. Thus, there is a discrepancy between the cellular effects of BCL6 inhibition mediated by compound 79-6 and by the specific siRNA against BCL6. One possible explanation for this complex observation may be the specificity of compound 79-6. This small molecule is able to block the BTB domain of BCL6, whereas the RB domain stays unaffected [9]. Therefore, such a partial and selective inhibition of the BCL6 molecule may be advantageous for its properties as an anti-tumor drug compared to total BCL6 downregulation by siRNA.

Only in MedB-1 cells the administration of current therapy components in combination with STAT6 downregulation did not lead to any advantage with respect to the drugs efficiency. This suggests a specific molecular background of this particular cell line. In fact, MedB-1 showed the highest amount of pSTAT6 within the three PMBL cell lines (pSTAT6 presence in MedB-1, K1106 and U-2940 is ~80%, ~25% and ~60%, respectively) [7] and harbours mutated STAT6, the function of which is still unclear [8]. Our results suggest not each PMBL may be suitable for the targeting of STAT6 in addition to current therapy. The identification of this particular PMBL subgroup needs further analysis and the mutations of STAT6 should be taken in account in this process.

The inactivation of STAT6 in PMBL by using selective JAK2 inhibitor, TG101348, reduced the growth of PMBL cells [7,10]. Taken into account that there are ongoing clinical trials regarding the use of TG101348 for patient treatment (myeloproliferative desease) [11], further knowledge about the role of mutated STAT6 in PMBL will be of interest and relevant for one third of PMBL [8] patients with respect to novel therapeutic approaches [2].

In sum, targeting of STAT6 / BCL6 by siRNA in two of three cell lines sensitizes the PMBL cells to the anti-tumor effects mediated by treatment with Doxo, RmAb, and Vincristin. Thus, the sensitisation of PMBLs to the current chemo-immunotherapy by the additional targeting of BCL6 / STAT6 may be useful and may help to reduce drugs toxicity and/or relapse in PMBL patients.

## MATERIAL AND METHODS

### Cell culture, transfection, and siRNA treatment

Three established human PMBL cell lines MedB-1, K1106, and U-2940 were maintained as described [7]. All cell lines were nucleofected with siRNA by using the manufacturer's dedicated protocol (Lonza, Cologne, Germany) and the following conditions: for MedB-1-buffer V (VCA1003; Lonza) program R-01; for K1106-buffer T (#VCA1002; Lonza) program N-20, for U-2940-buffer T, program X-05. Cells (4x10^6^) were transfected with 0.2nmol/sample of control siRNA (#VC300A2; Sigma-Aldrich, Taufkirchen, Germany) or siRNA targeting STAT6 (#STAT6VHS41762, Life Technologies, Darmstadt, Germany) or BCL6 (#BCL-6HSS100968, Life Technologies). After the incubation for 72h several components of current R-CHOP were added or not to the cells.

### Western blot, band-shift, and super-shift assays

Western blots were performed as described [8] by using 10μg total extracts per lane and antibodies against BCL6 (N3 #sc-858, Santa Cruz, Heidelberg, Germany); STAT6 (M-200 #sc-1698, Santa Cruz); β-Actin (Sigma).

### Drug treatment and cell viability assays

PMBL cell lines were treated for 24h with Doxo (0,1ng/ml), VC (1μg/ml) and RmAB (1μg/ml). According to manufacture's protocol the cell viability analysis was performed using the CellTiter-Glo® Luminescent (Promega, Mannheim, Germany.

T-tests were used for statistical comparisons and the statistical significance was defined as *p* <0.05.
